# Synovial single-cell heterogeneity, zonation and interactions: a patchwork of effectors in arthritis

**DOI:** 10.1093/rheumatology/keab721

**Published:** 2021-09-24

**Authors:** Barbora Schonfeldova, Kristina Zec, Irina A Udalova

**Affiliations:** The Kennedy Institute of Rheumatology, Nuffield Department of Orthopaedics, Rheumatology and Musculoskeletal Science, University of Oxford, Oxford, UK

**Keywords:** RA, synovium, single-cell transcriptomics, cellular localization, experimental arthritis

## Abstract

Despite extensive research, there is still no treatment that would lead to remission in all patients with rheumatoid arthritis as our understanding of the affected site, the synovium, is still incomplete. Recently, single-cell technologies helped to decipher the cellular heterogeneity of the synovium; however, certain synovial cell populations, such as endothelial cells or peripheral neurons, remain to be profiled on a single-cell level. Furthermore, associations between certain cellular states and inflammation were found; whether these cells cause the inflammation remains to be answered. Similarly, cellular zonation and interactions between individual effectors in the synovium are yet to be fully determined. A deeper understanding of cell signalling and interactions in the synovium is crucial for a better design of therapeutics with the goal of complete remission in all patients.

Rheumatology key messages

- Single-cell technologies have identified the diverse immune and structural cell composition of the synovium.
- Many human synovial cell populations appear to have their counterparts in the murine synovium.
- The cellular localisation and cell–cell interactions in the synovium are yet to be fully deciphered.

## Introduction

RA is a chronic inflammatory disease that mainly affects the synovium that nourishes and supports the joint during homeostasis (disease pathology was reviewed, for example, in [1, 2]). A better understanding of synovial biology could help in identifying novel drugable targets for the pursuit of achieving sustained remission or, better yet, finding a cure for RA [3]. RA environment and its biomarkers were originally studied indirectly in peripheral blood or synovial fluid; however, better synovial sampling through arthroscopic or ultrasonography-guided biopsies allowed for immunohistochemical and biochemical profiling of the synovial composition leading to better understanding of the disease site (reviewed in [4]). Still, profiling cellular heterogeneity, localisation, and interactions in the synovium and integrating those data is the way forward in disentangling the underlying mechanisms of inflammation as well as those underlying resolution.

Single-cell technologies allowed for deep characterisation of the synovial tissue heterogeneity; however, recent reviews have been almost exclusively focussed on fibroblasts, macrophages/monocytes, T cells and B cells and omitted other structural and supporting cells such as endothelial cells or peripheral neurons. In this review, we aim to comprehensively describe synovial cell populations, their activation states and association with synovial pathology, as well as identify knowledge gaps and populations remaining to be profiled. Subsequently, we will discuss the positioning (zonation) of individual subpopulations of cells in the synovium and how the single-cell profiling can assist in discovering the localisation of those cells. Finally, we will provide a summary of the known intercellular interactions as well as identify which tools can be used to predict and confirm further signalling pathways.

## Cellular heterogeneity in the synovium

### Methods to study single-cell heterogeneity

**Fig. 1:**
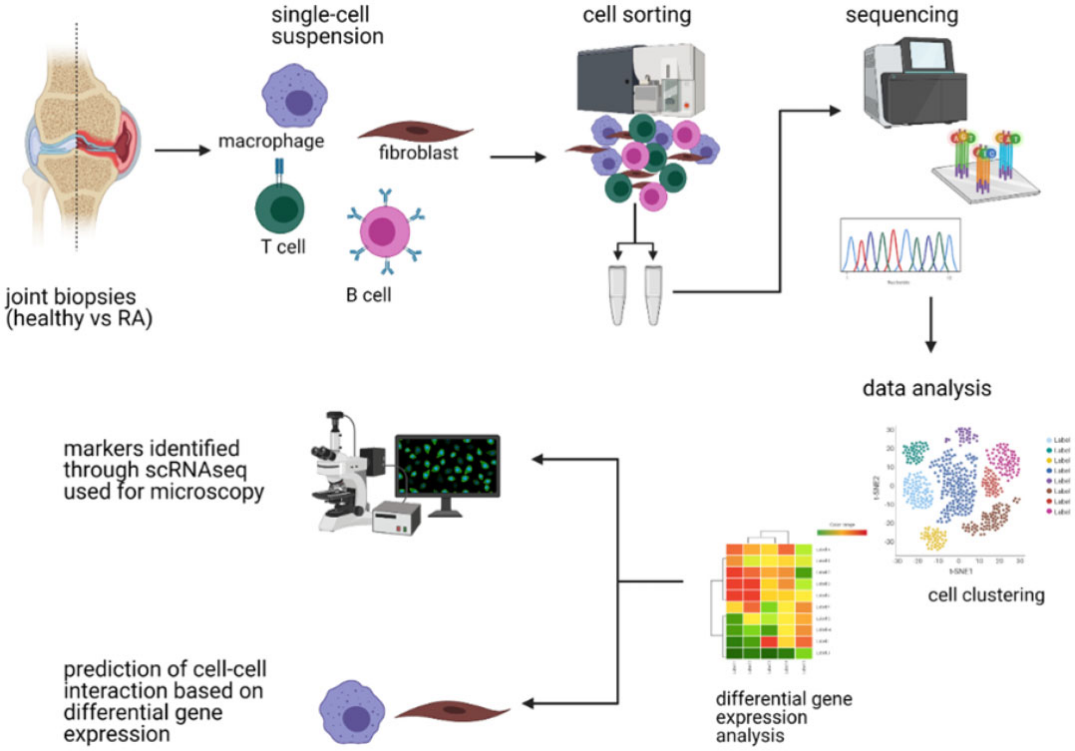
Simplified workflow of a sequencing experiment The sample acquisition (e.g. human or murine joint biopsies) is followed by the processing of the sample into a single-cell suspension. Population of interest is then sorted by FACS and prepared for sequencing (e.g. barcoding the mRNA to identify cell source, reverse transcription, preparation of cDNA library). Raw sequencing data need to be pre-processed and analysed. The findings from single-cell RNA sequencing experiments should be experimentally validated. Figure created with BioRender.com.

The field of single-cell sequencing is rapidly growing, and several new methods have been developed to facilitate the in-depth characterisation of different cell types. These novel technologies, their applications, and their integration into existing research frameworks have been recently reviewed (for example in [5–7]). Single-cell transcriptomics has been extensively used to profile synovial tissue and infer the cellular function and contribution to inflammation (Fig.  1). Several methods such as Smart-seq2 [8] or 10X Genomics [9] are in routine use and others, such as the low-cost microfluidic instrument, were also reported for profiling the synovium in RA [10]. However, some challenges remain in the use of these technologies (reviewed in [11]). Following the best practices during the analysis is crucial to ensure that results are valid and reproducible [12].

### Immune cells

#### Mononuclear phagocytic cells

Mononuclear phagocytes are important contributors to both homeostasis and inflammation. The role of synovial macrophages in the context of RA has been extensively studied in patients as well as in animals (recent reviews: [13–15]). Previously, tissue-macrophages were found to restrict excessive neutrophil recruitment [16]; however, recent single-cell experiments revealed substantial heterogeneity among synovial myeloid cells in both mice and humans [17–20].

**Fig. 2:**
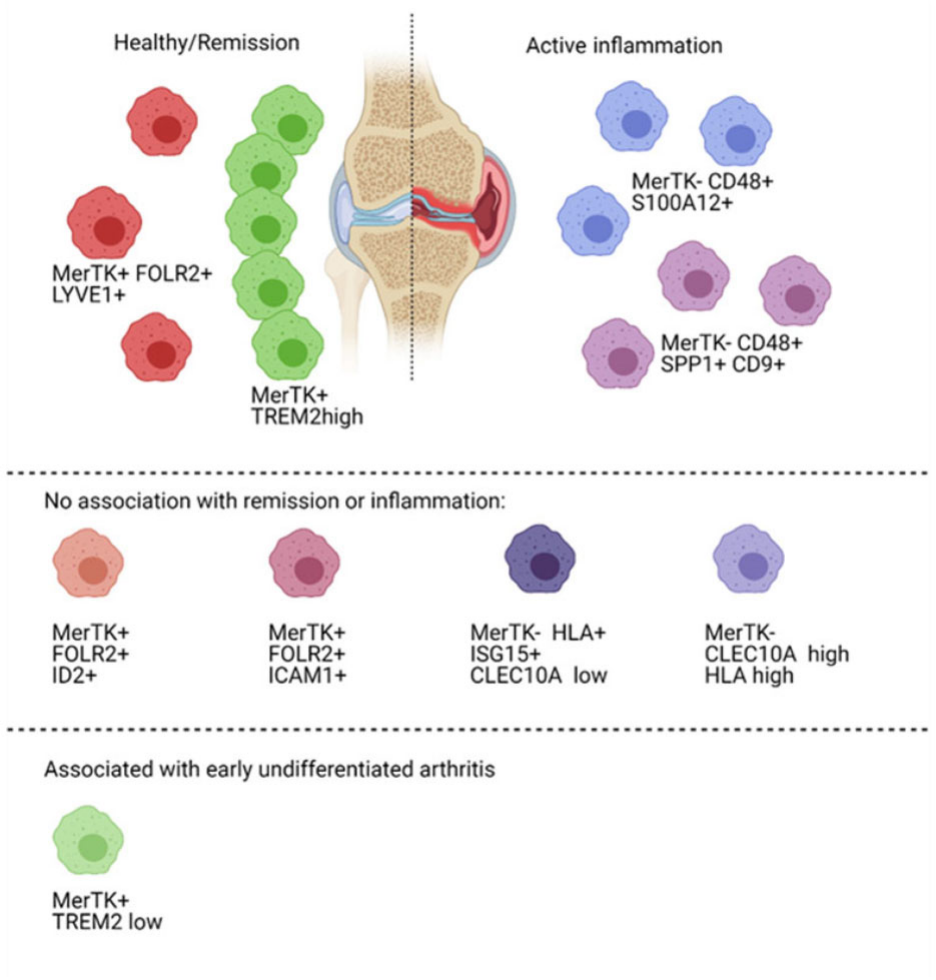
Macrophage populations identified in human synovium (based on [17]) Macrophage populations characterised as MerTK^pos^FOLR2^pos+^LYVE1^pos^ or MerTK^pos^TREM2^high^ were associated with remission, while MerTK^neg^CD48^pos^S100A12^pos^ or MerTK^neg^CD48^pos^SPP1^pos^CD9^pos^ macrophages were preferentially found in actively inflamed synovium. Other populations had no clear association with either remission or inflammation. Interestingly, an increased number of MerTK^pos^TREM2^low^ macrophages was found in early undifferentiated arthritis. Figure created with BioRender.com.

Single-cell profiling of synovial macrophages in healthy controls, treatment-naïve/-resistant patients with RA, and patients in sustained remission revealed nine distinct subpopulations (Table  1, Fig.  2) [17]. The association of these subsets to inflammation *vs* remission was largely determined by the expression of MER tyrosine kinase (*MERTK*); MerTK^pos^ macrophages were more abundant in stable remission and healthy controls, while MerTK^neg^ subset was relatively scarce in healthy synovium and over-abundant in active RA [17]. Similarly, *MERTK*-expressing macrophages were upregulated with anti-inflammatory medication in RA patients [20] and expression of *Mertk* itself was elevated during resolution of inflammation in a murine model of arthritis [21]. Furthermore, the ratio of MerTK^pos^ to MerTK^neg^ macrophages was also found to be predictive of flares’ occurrences [17] showing MerTK as a potential predictive tool for clinicians. Conversely, MerTK^neg^ subpopulations show similarity to previously identified pro-inflammatory macrophages [17, 19]. Another pro-inflammatory population was identified in RA patients, marked by high expression of heparin binding EGF-like growth factor (HBEGF), that interacted with synovial fibroblasts and was predicted to increase fibroblast invasiveness and neutrophil recruitment [20].

**Table 1:** Synovial myeloid cell heterogeneity (based on [17])

Markers defining this population			Associated with inflammation/ remission	Possible function, associated expression profile	Mouse equivalent (Culemann *et al.* 2019)
MerTK^pos^	TREM2^pos^	TREM2^low^	Increased in early undifferentiated arthrits	May represent early activated TREM2^high^	
		TREM2^high^	Remission	Restraining inflammation; expression associated with complement and defensin pathways, Treg differentiation, restriction of effector T-cell function, scavenger receptor etc.	lining Trem2^pos^ Cx3cr1^pos^
	FOLR2^pos^	ID2^pos^	Similar abundance in all conditions	May represent *in situ* precursors of resident macrophages	
		LYVE1^pos^	Remission	Immuno-regulation; gene expression associated with collagen turnover, antiprotease enzymes, coagulation factors and regulators of vascular endothelial growth factor	RELMα+ sublining mph
		ICAM1^pos^	Similar abundance in all conditions	May represent first-line defence against pathogens; high expression of proinflammatory cytokine genes	
MerTK^neg^	HLA^pos^	ISG15^pos^ CLEC10A^low^		Interferon signalling	
		CLEC10A^high^ HLA^high^	Similar abundance in all conditions	May represent synovial tissue-resident antigen-presenting cells	
	CD48^pos^	S100A12^pos^	Inflammation	Proinflammatory phenotype; expression of alarmin S100A9 correlated with disease severity	
		SPP1^pos^ CD9^pos^	Inflammation	Proinflammatory phenotype; osteopontin expression correlated with disease severity	

Similarly, macrophages in a murine RA model, serum transfer-induced arthritis (STIA), were identified as a heterogeneous population displaying dynamic changes in their respective abundances over the first 5 days of the disease [18]. This was largely caused by the macrophage infiltration during disease development. At the initial stage, seven subpopulations of macrophages were identified: C-X3-C motif chemokine receptor 1 (CX_3_CR1)^+^ lining, resistin-like alpha (RELM-α)^+^ interstitial, aquaporin 1 (AQP1)^+^ interstitial, C-C chemokine receptor type 2 (CCR2)^+^ infiltrating, major histocompatibility complex class II (MHCII)^+^ interstitial, stathmin 1 (STMN1)^+^ proliferating macrophages and lymphocyte antigen 6 complex, locus C2 (LY6C2)^+^ monocytes [18]. CX_3_CR1^+^ lining macrophages expressed genes associated with immune-regulation, clearance of apoptotic cells, and barrier formation [18], suggesting important homeostatic function within the synovium.

Monocytes are important for the development of STIA; their absence is associated with lower leukocyte recruitment and restriction of inflammation [21]. Furthermore, compared with OA samples that are not defined by immune cell abundance, RA biopsies have a strong myeloid expression signature [22]. Thus, profiling of myeloid cells in inflammatory arthritis contributes to a better understanding of this disease. Synovial macrophage subpopulations, their associations with pathology, predicted functions and comparison between humans and mice are summarized in Table  1.

#### Granulocytes

Due to the high levels of intracellular RNases, granulocytes are difficult to profile by transcriptomic approaches. Despite those difficulties, several studies were able to profile heterogeneity of granulocytes, mainly neutrophils, during both homeostasis and inflammation [23, 24]. Such transcriptomic advances should be applied to investigate the synovial granulocytes considering the importance of neutrophils at the onset of arthritis in the murine models [25–27]. Diversification of neutrophil phenotypes could be of particular interest as low-density neutrophils have been recently identified to correlate with pathology in multiple inflammatory diseases (for example [28–30]). Interestingly, type 2 responses, including an increased number of eosinophils within the joint, were found protective in murine models of arthritis [31, 32] and were mediated by IL-4/IL-13-induced signal transducer and activator of transcription 6 (STAT6) activation [31]. Granulocyte-associated gene patterns, such as markers of phagocytosis, pattern recognition receptor and complement activation, were apparent in synovial samples of RA patients [22, 33]. Therefore, closer profiling of granulocytes within the synovium would be beneficial to determine their contribution to inflammatory arthritis.

#### B lymphocytes

Autoantibodies against IgG antibodies (RF) and citrullinated proteins (ACPA) are characteristic of RA in certain patients but their presence is not universal, a phenomenon indicative of heterogeneous B-cell responses. Gene patterns associated with classical B-cell function have been detected only in synovial tissue of patients with RA, not those with OA [22]. Moreover, B-cell activation and differentiation-associated gene signature was predictive of increased bone erosion [33].

Single-cell characterisation of B cells in the synovium of patients with OA or RA revealed four distinct subpopulations of B cells: naïve B cells (called SC-B1), IGHG+CD27+ memory B cells (SC-B2), autoimmune-associated B cells (SC-B3) and plasmablasts (SC-B4) [19]. The SC-B3 cluster had an elevated expression of markers associated with B-cell activation, interferon stimulation and autoimmunity, and was overabundant in inflamed synovium compared with OA controls [19]. A subset of receptor activator of nuclear factor kappa-Β ligand (RANKL)-producing B cells was also identified in the synovial fluid and patient tissue [34]. Further validation of the functionality of these cells will be necessary to confirm their proinflammatory and autoimmune role in RA. In-depth B-cell profiling may be valuable to a subset of patients; however, as patients manifest with discrete levels of lymphocyte infiltration [33, 35, 36], the effectivity of drugs targeting B-cell activation may be limited.

#### T lymphocytes

T cells form an important part of the adaptive immune system and transcripts associated with T cell function were detected in synovial tissue isolated from patients with RA but not in OA, suggesting their role in inflammation [22]. Conversely, Th2 responses were found to be protective in murine models of RA [31, 32], a discrepancy potentially explained by synovial T-cell heterogeneity [19, 33, 37].

ScRNAseq of the synovial tissue isolated from patients with either OA or RA revealed three subpopulations of CD4+ [CCR7+ (called SC-T1), forkhead box P3 (FOXP3)+ regulatory (SC-T2), PDCD1+ peripheral helper (T_PH_) and follicular helper T cells (T_FH_) (SC-T3)] and three subpopulations of CD8+ T cells [GZMK+ (SC-T4), GNLY+GZMB+ cytotoxic (SC-T5), GZMK+GZMB+ T cells (SC-T6)] [19]. SC-T3 T_PH_ and T_FH_ cells were found to produce high levels of a chemokine C-X-C motif chemokine ligand 13 (*CXCL13*), inhibitory receptors [T cell immunoreceptor with Ig and ITIM domains (*TIGIT*)*,* cytotoxic T-lymphocyte-associated protein 4 (*CTLA4*)] and were associated with inflammation in the synovium [19]. The population of T_PH_ cells, characterised as PD1^hi^CXCR5^-^CD4^+^, was found to be expanded in the joints of seropositive RA patients and supported B-cell differentiation and antibody production [37]. Similarly, PD-1^high^ T_PH_ cells producing *PDCD1, TIGIT* and *CXCR6* were associated with strong inflammation in treatment-naïve patients with RA [33]. Overall, T_PH_ cells may represent a pathological player in RA.

Studies in psoriatic arthritis showed that expanded T cells are likely retained in the synovium by an increased expression of *CXCR3* or *CXCR6* whose ligands CXCL10 and CXCL9 or CXCL16 respectively are present in an increased level in the synovial fluid during the disease [38, 39]. Although untested, similar retention could apply to PD-1^high^ T_PH_ in RA as they also express an increased level of *CXCR6* [33]. Inhibiting the retention of these proinflammatory T cells may present a potential therapeutic target; however, further research is required to validate T-cell relationships to pathology identified by single-cell experiments.

### Structural cells

#### Tissue maintenance: fibroblasts

Stromal cells including fibroblasts are important contributors to synovial homeostasis and have a substantial role in the pathology of RA (recently reviewed in [40–42]). Recent single-cell studies revealed vast heterogeneity among this population and the differential role of distinct fibroblast clusters in RA pathogenesis [19, 43–45].

**Fig. 3:**
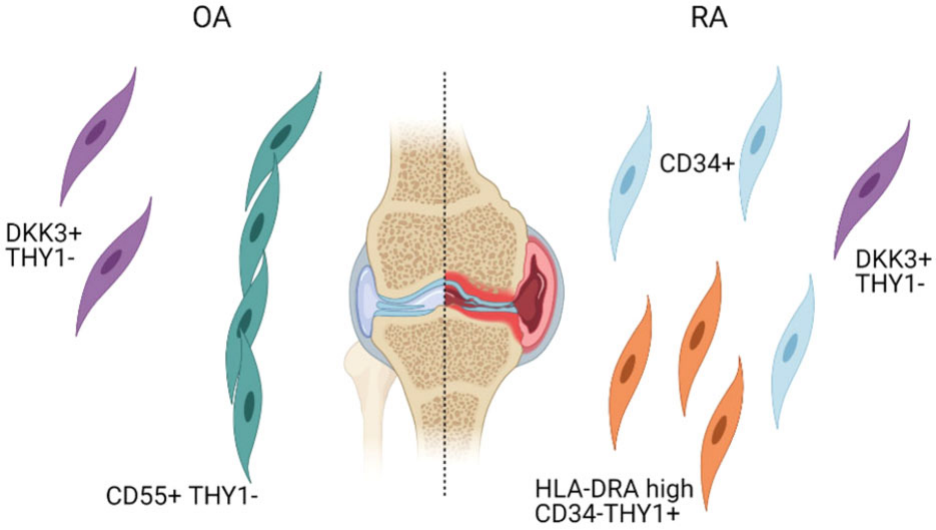
Fibroblast populations identified in human synovium (based on [19]) CD55+THY1- lining fibroblast were found to be associated with OA while CD34+ and HLA-DRA^hi^CD34-THY1+ sublining fibroblasts were preferentially found in RA. Population of sublining fibroblasts defined as DKK3+THY1- was not clearly associated with either. Figure created with BioRender.com.

ScRNAseq profiling of synovial fibroblasts isolated from patients with RA or OA revealed three major subsets: CD34-THY1-, CD34-THY1+ and CD34+ [43] (Fig.  3). CD34-THY1+ were more abundant in RA than in OA, positively correlated with leukocyte infiltration, histological synovitis and hypertrophy [43]. Furthermore, CD34-THY1+ fibroblasts expressed factors that drive osteoclastogenesis and triggered the generation of triiodothyronine receptor auxiliary protein (TRAP)+ osteoclasts *in vitro* [43]. CD34+ fibroblasts were characterized by high expression of interleukin 6 (*IL6*)*, CXCL12,* C-C motif chemokine ligand 2 (*CCL2). In vitro* experiments confirmed their ability to recruit a high number of peripheral blood monocytes [43].

A similar analysis of synovial tissue from RA and OA patients by single-cell technologies identified four fibroblast subpopulations: CD34+ sublining fibroblasts (termed SC-F1), human leukocyte antigen (HLA)-DRA^hi^ sublining fibroblasts (SC-F2), Dickkopf WNT signalling pathway inhibitor 3 (DKK3+) sublining fibroblasts (SC-F3) and CD55+ lining fibroblasts (SC-F4) [19]. The HLA-DRA^hi^ population of fibroblasts (SC-F2) [19] seems to correspond to the previously defined inflammatory CD34-THY1+ fibroblasts [43]. They expressed genes associated with antigen presentation, IFNγ signalling, high levels of *IL6* and *CXCL12*, and were overabundant in leukocyte-rich RA samples compared with leukocyte-poor RA and OA samples [19].

Furthermore, RA patients have a higher number of fibroblast activation protein alpha (FAPα)+ fibroblasts compared with patients without active inflammation [44]. Also, FAPα+ fibroblasts were significantly associated with inflammation, leukocyte infiltration, joint damage and pannus formation in STIA [44]. In this murine model, scRNA-sequencing identified five distinct fibroblast populations [44]. FAPα+THY1+ were found to represent sublining fibroblasts which increased the severity and persistence of inflammation, while FAPα+THY1- fibroblasts were located to the synovial lining and had a role in bone and cartilage destruction [44].

Overall, sublining THY1+ fibroblasts are expanded in both RA and animal models of this disease and associate with increased leukocyte infiltration [19, 43–45] representing a potential therapeutic target. The comparison of murine and human synovial fibroblasts, their identifying markers and predicted functions are summarized in Table  2.

**Table 2:** Synovial fibroblast heterogeneity [19]

Markers defining this population	Name in Zhang *et al.* 2019	Associated with RA/OA	Possible function, associated expression profile	Position in the synovium	Mouse equivalent (Croft *et al.* 2019)
CD34+	SC-F1	Associated with inflammation in RA	Expression of *IL6, CXCL12, CCL2*; potential role in monocyte recruitment (Mizoguchi *et al.* 2018)	Sublining: observed in both superficial and deeper sublining areas (Mizoguchi *et al.* 2018)	
HLA-DRA^hi^	SC-F2	Associated with inflammation in RA	MHC II antigen presentation and IFNγ signalling; production of proinflammatory cytokines; potential role in leukocyte infiltration (Mizoguchi *et al.* 2018)	Sublining	FAPα+THY1+CD34- (STIA *Col11a1+* F1 population)
DKK3	SC-F3	No association found	Identified as a novel subpopulation with high expression of *DKK3, CAMD1, COL8A2*	Sublining	
CD55	SC-F4	OA associated	murine FAPα+THY- lining fibroblast may be potentially associated with bone and cartilage erosion (Croft *et al.* 2019)	Lining	Similarity to FAPα+THY- lining fibroblasts, (STIA F5 lining population)

#### Vascularisation: endothelial cells

Increased vascular permeability is a feature of inflammatory models of arthritis and endothelial dysfunction is a common complication of RA (reviewed, for example, in [27, 46, 47]). Synovial endothelial cells, therefore, seem to have an impact on the propagation of the inflammation.

Endothelial cells are a heterogeneous population in other tissues with organ-specific characteristics supporting the needs of the local microenvironment [48–52]. These differences are apparent in ECs’ appearance, morphology and transcriptional profile [48]. Both human and murine endothelial cells even show sex-specific transcriptome differences across organs such as the aorta, heart and lung [49, 53].

Human synovial arterial (PODXL+) and venous [Duffy blood group, atypical chemokine receptor (DARC+)] endothelial cells were found to contribute differential cues to the synovial microenvironment [45]. Arterial endothelial cells, not venous ones, support the differentiation of synovial sublining fibroblasts [45]. Endothelial cells also assist local cell functionality in other organs such as promoting hepatocyte survival and albumin production in the liver [48]. Furthermore, capillaries traversing cortical bone from the bone marrow have been recently identified [54]; however, their contribution to the synovial environment remains to be established.

Regrettably, endothelial heterogeneity in the joint has not been sufficiently studied. Considering the importance of vascular permeability in the immunopathology of RA, it would be interesting to investigate endothelial cell functions of the synovium in steady-state and inflammation. Understanding synovial endothelium could lead to the development of novel therapeutics that target vascular permeability and oedema.

#### Innervation: peripheral nervous system

Joint-associated pain is common to both RA and OA [1, 2, 55, 56]. In a murine model of OA, osteoclasts induced axonal growth in the subchondral bone by secreting netrin-1, which was predicted to mediate increased sensitivity of the peripheral nervous system and pain perception [57]. As aberrant innervation of the cartilage is seen in an animal model of inflammatory arthritis [58], similar effector pathways could mediate joint pain in RA as well. Changes in the dorsal horn of the central nervous system in inflammatory arthritis were also detected [58]. Furthermore, the loss of sensory innervation in the joint was found to be protective in a patient with psoriatic arthritis [59]. This effect of protective sensory denervation was confirmed in an animal model of inflammatory arthritis [59]. Of note, partial denervation of the wrist resulted in self-reported improvement of pain perception in patients with inflammatory arthritis [60].

The peripheral nervous system was found to be formed by a highly heterogeneous population of cells (among many, for example, [61–64]). In addition to heterogeneous populations of myelinating and non-myelinating Schwann cells, neural-associated endothelial cells, fibroblasts and leukocytes were also identified [64]. Leukocytes of the peripheral nervous system consist of CX3CR1-expressing myeloid cells with CXCL4+ macrophages, also identified in the mouse brain [64, 65]. Changes in the populations of cells associated with peripheral nerves were observed in an animal model of spontaneous chronic peripheral neuritis [64]. Similarly, sensory and sympathetic neurons innervating the draining lymph nodes were found to be highly heterogeneous, able to communicate with surrounding stromal and immune cells and expand after toll-like receptor 4 stimulation [63].

In conclusion, the heterogeneity of synovial innervation and its contribution to the pathology of RA remain incompletely understood. However, both peripheral and central nervous system seem to be altered in inflammatory arthritis [58], providing the rationale for the development of pain-modifying drugs that interfere with neural effector pathways to improve the quality of life of patients with both RA and OA [66].

## Cellular zonation in the synovium

### Methods to study cellular zonation

Fluorescence imaging and its implication for deciphering organ function have been recently reviewed [67]. Advances in confocal microscopy, for example by the addition of Airyscan detectors, and in 3D imaging were beneficial for the field of rheumatology as well. However, spatiotemporal localisation of different subsets of immune and structural cells within the synovium has been difficult due to the lack of markers characterizing different subpopulations. As described in the previous section, for most synovial cells, single-cell transcriptomics helped to overcome this issue by providing a characterisation of differentially expressed genes across populations. Furthermore, the recent development of spatial transcriptomics such as sequential fluorescence *in situ* hybridization [68], Slide-seq [69] or commercially available 10X Genomics’ Visium and nanoString is becoming instrumental to further understand the spatiotemporal changes in the synovium. Spatial transcriptomics has been used already to study cellular niches and networks in Alzheimer’s disease [70] or carcinomas [71] and very recently also to uncover signalling and localisation of infiltrating leukocytes in RA [35, 36].

### Immune cells

Two major interstitial macrophage niches were recently identified in mice by combining transcriptional profiling and fluorescence imaging, namely vasculature and nerve-associated macrophages [72]. Lyve1^lo^MHCII^hi^ tissue-resident macrophages expressed higher levels of genes associated with antigen presentation and were more closely associated with peripheral nerves [72]. Conversely, Lyve1^hi^MHCII^lo^ macrophages were found to contribute to tissue repair and resided close to the vasculature [72]. These macrophage populations were identified in murine lung, fat, heart and dermis, but also in several human tissues [72]. We compared the expression of markers defining these two interstitial populations to the murine synovial macrophages from Culemann *et al.* [18] and found that synovial RELMα+ sublining macrophages resembled Lyve1^hi^MHCII^lo^, while synovial MHCII^+^ interstitial macrophages were more like Lyve1^lo^MHCII^hi^. This suggests that RELMα+ sublining macrophages are localized closer to the blood vessels and MHCII^+^ interstitial macrophages near the synovial innervation. This hypothesis is further supported by the resemblance of synovial RELMα+ macrophages to human MerTK^pos^FOLR2^pos^ LYVE1^pos^ macrophages, which produce high levels of perivascular marker lymphatic vessel endothelial hyaluronan receptor 1 (LYVE1) and express markers associated with regulation of vascular endothelial growth factor [17]. However, the exact positioning of synovial interstitial macrophages requires further investigation by imaging technologies.

CX_3_CR1^+^ lining macrophages were identified as a dense protective barrier which is Csf1R^-^ and gets replenished by Csf1R^+^ sublining macrophages [18]. Based on published confocal microscopy images by Culemann *et al.* [18] and our independent observations, lining macrophages form the uppermost lining layer; however, it is not a dense layer similar to epithelial cells, but it can be rather fragmented.

Finally, as the peripheral helper T cells were found to have a pathological role in B-cell activation in inflammatory arthritis [19, 33, 37], these cells need to be localized close to each other. By immunohistochemistry, T_PH_ cells in synovial lymphoid aggregates were found in proximity to B cells [37]. In ∼40% of patients with RA, leukocyte infiltrates manifest as tertiary lymphoid organ-like structure (reviewed, for example, in [73, 74]). B cells and T cells seem to have an organized positioning within these structures with CCL21+ cells on the inside and marginal zone B and B1 cell specific protein (MZB1)+ plasma cells on the outside [35]. Vickovic *et al.* [35] hypothesized that tertiary lymphoid organ-like structures retain newly recruited leukocytes; however, the positioning of these structures in respect to the vasculature or other synovial immune and structural cells is unknown.

### Structural cells

Similar to immune cells, different subsets of fibroblasts localize to different regions of the synovium [19, 43–45]. CD34-THY1+ fibroblasts were found to reside in the sublining where they surrounded the capillaries [43]. This layer was a few cells thin in OA patients and expanded in the synovium isolated from patients with active inflammation [43]. CD34+ fibroblasts could also be observed in the sublining layer, scattered across both deep and superficial interstitium [43]. Finally, CD34-THY1- produced lubricin and formed the synovial lining layer [19, 43, 44]. Overall, THY1 expression determines sublining populations of fibroblasts [19, 43–45]. Furthermore, the distance from endothelial cells seems to affect the phenotype of fibroblasts; cells closest to the vasculature express high levels of THY1 and cells furthest express high levels of lubricin [45]. In conclusion, local microenvironment cues are crucial for cell positioning, identity and function.

## Cell–cell interactions in the synovium

### Methods to study cellular interactions

Possible methods of how cellular interactions can be studied based on gene expression were nicely summarized in a recent review [75]. In brief, a variety of methods have been developed such as NicheNet [76], SoptSC [77, 78], CellPhoneDB [79], CellChat [80], and many others. Each method is using a different algorithm for assessing cell–cell interactions, such as using differential gene expression between and within cell clusters to search for ligand-receptor pairs. These approaches also use different data sources to access ligand-receptor pairs; for example, NicheNet is using KEGG as its main data source [76]. The assessment of individual strengths and weaknesses of the available methods is not the subject of this review. Nevertheless, as emphasized in the recent review [75], these approaches should be considered mainly for hypothesis generation and require subsequent experimental validation. Additionally, spatial transcriptomics can help with understanding cellular interactions by providing site-specific expression profile of the synovium [35, 36].

### Identified cellular interactions in the synovium

**Fig. 4:**
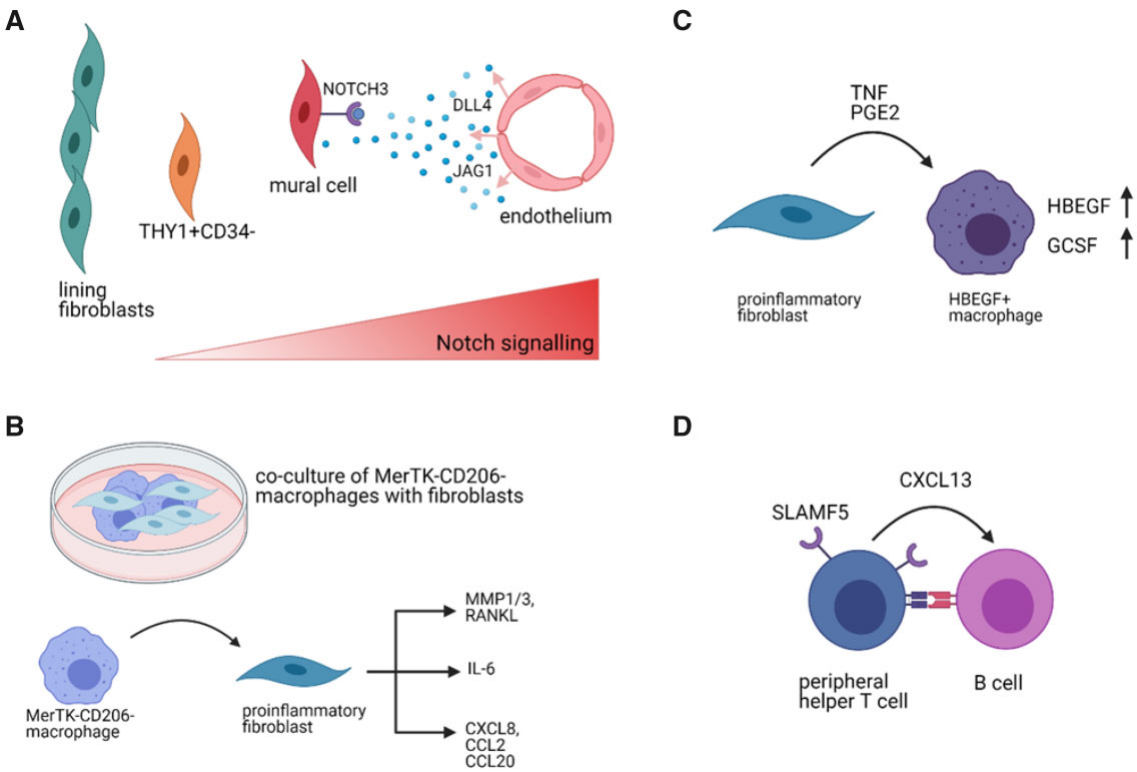
Examples of interactions identified in human or murine synovium **(A)** Arterial endothelial cells were found to release NOTCH3 ligands DLL4 and JAG1 that bind NOTCH3 receptor on mural cells and sublining fibroblasts. The Notch signalling gradient shapes the identity of fibroblasts where fibroblasts closer to the endothelium will develop into THY1+CD34- fibroblasts and cells further from it will become THY1- lining fibroblasts. (Based on [45]) **(B)** Co-culture of proinflammatory MerTK^neg^CD206^neg^ macrophages with fibroblasts caused the fibroblasts to adopt a proinflammatory phenotype and secrete bone and cartilage destructing mediators (MMP1/3, RANKL) and inflammatory cytokines (IL6) or chemokines (CXCL8, CCL2, CCL20) [17]. **(C)** Proinflammatory fibroblasts were found to secrete TNF or PGE2, which induce expression of HBEGF and GCSF in macrophages, inducing pro-inflammatory phenotype [20]. **(D)** Peripheral helper T cells and B cells communicate in the synovium through multiple pathways, for example via secretion of CXCL13 by T cells [19, 37, 81] or via upregulation of SLAMF5 [37]. Figure created with BioRender.com.

The ligand-receptor analysis was used to identify the Notch signalling axis between fibroblasts and endothelial cells, which shapes fibroblast identity in the synovium [45]. Based on this analysis, Wei *et al.* [45] identified that synovial arterial endothelial cells express Notch ligands jagged canonical Notch ligand 1 (*JAG1*) and delta-like canonical Notch ligand 4 (*DLL4*), which interact with NOTCH3 on surrounding mural cells and fibroblasts (Fig.  4A). Furthermore, the endothelial cells seemed to direct the differentiation of *THY1+* sublining fibroblast, which was confirmed in mixed synovial organoids [45].

Fibroblasts were also found to be affected by synovial macrophages in an *in vitro* co-culture [17]. MerTK^neg^ synovial macrophages induced an increased expression of mediators of bone and cartilage destruction, inflammation and leukocyte recruitment in fibroblasts [17] (Fig.  4B). Vice versa, in the resolution phase of RA, THY1^pos^CXCL14^pos^ sublining fibroblasts were found to abundantly express an activator of MerTK, growth arrest specific 6 (GAS6) [17]. MerTK was previously found to be significantly associated with the resolution of inflammation [17, 20, 21].

Synovial fibroblasts further impact the transcriptome of macrophages by secreting pro-inflammatory factors such as TNF and prostaglandin E2 (PGE2) and support their development into inflammatory HBEGF^+^ macrophages [20] (Fig.  4C). Medications, such as leflunomide, dexamethasone, naproxen and triple therapy could inhibit the fibroblast induced HBEGF^+^ macrophage polarization [20]. For example, naproxen functionally blocked the prostaglandin generation and in turn abrogated HBEGF mRNA and granulocyte colony-stimulating factor (GCSF) protein production [20], showing the importance of both cellular heterogeneity and interactions in understanding the molecular action of anti-inflammatory drugs.

Usage of spatial transcriptomics [81] revealed an upregulated expression of genes such as lymphotoxin beta (*LTB*), *CCL19*, *CXCL13*, *CD52*, membrane spanning 4-domains A1 (*MS4A1*) and *CD79A*, which are associated with T-cell and B-cell function and crosstalk [35, 36]. Especially, CXCL13 expression by T_PH_ to activate B cells is of interest as it is confirmed in other sequencing studies [19, 37]. Furthermore, signalling lymphocytic activation molecule 5 (SLAMF5), required for T-cell and B-cell interaction, was upregulated on peripheral and follicular helper T cells and a blockade of SLAMF5 abrogated PD1^hi^CXCR5^-^CD4^+^ T-cell effect on B cells in an *in vitro* co-culture [37] suggesting an additional therapeutic target (Fig.  4D). However, this approach would most likely be restricted to seropositive patients. Additionally, patients with inflammatory arthritis have a varying degree of B-cell and T-cell infiltration [33], further complicating this approach.

## Conclusion

**Fig. 5:**
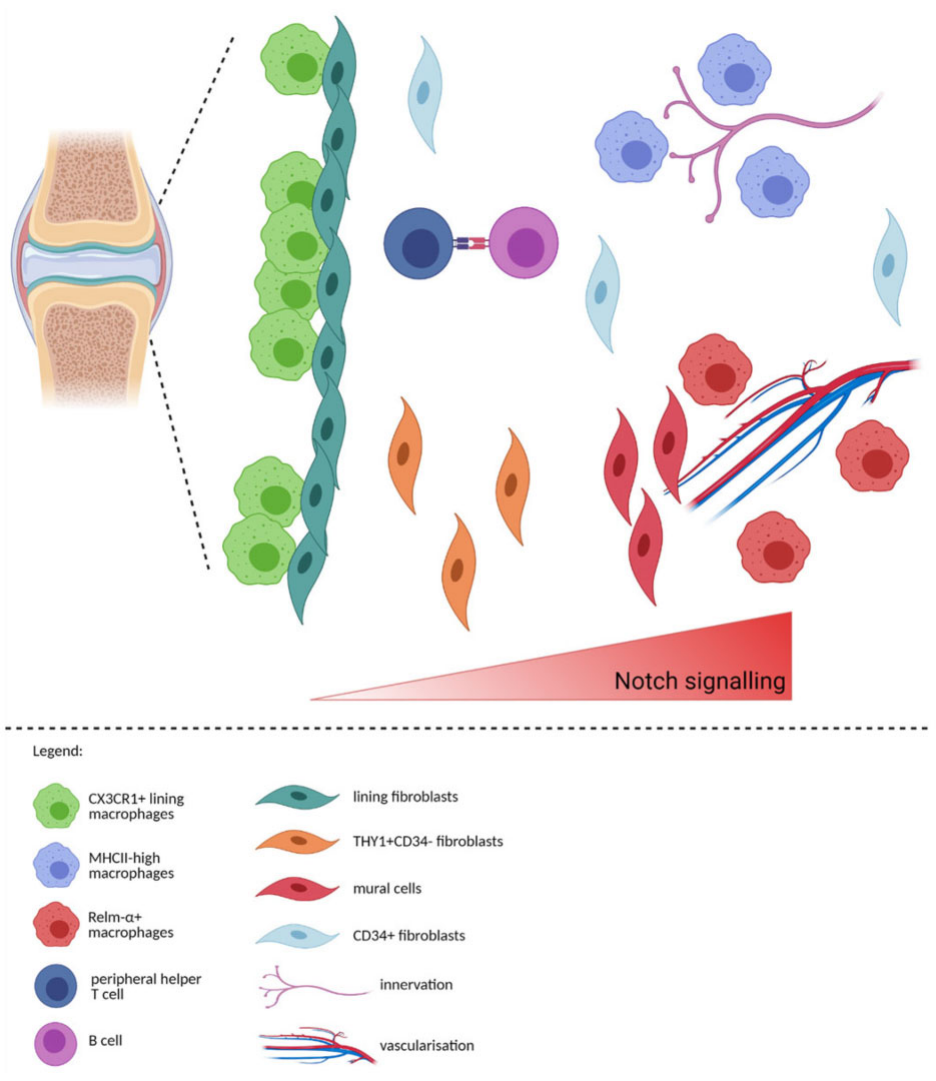
Prediction of the zonation of immune and structural cells in the synovium CX_3_CR1+ lining macrophages and lining fibroblast create the ‘barrier’ layer of the synovium [18, 19]. Based on our analysis, MHCII^high^ macrophages could be positioned near the innervation, while RELM-α+ macrophages could be located near the vasculature, similarly to the lung interstitium [72]. Notch signalling axis is instructing the positioning of fibroblasts with mural cells closest to the vasculature, THY1+CD34- fibroblasts near the vasculature and mural cells with THY1-CD34- lining fibroblast at the opposing end [43, 45]. CD34+ fibroblasts are positioned both in the immediate sublining and deep interstitium [43]. Figure created with BioRender.com.

Recent advances in single-cell technologies allowed for deeper profiling of synovial cells, identification of their respective markers, activation states and provided predictions of their functionality (Fig.  5). Studying cellular organization is virtually impossible without an appreciation of the characteristic cellular markers; therefore, we envisage that the field of rheumatology will soon be enriched with many studies profiling cellular zonation in the synovium. The spatial characterisation will further uncover which cells are likely to interact with each other, leading to a better understanding of signalling pathways in the synovium both in the steady-state and during inflammation. Single-cell transcriptomics already led to the development of computational methods allowing for the prediction of ligand-receptor interactomes but, as mentioned before, these tools should be used for hypothesis generation and subsequently experimentally verified. Apart from cellular heterogeneity, the extracellular matrix composition, function and localisation creating unique niches was recently reviewed as well [82]. Thus, the interactions between synovial cells and the extracellular matrix should also be considered to fully comprehend the maintenance and function of this complex tissue. Furthermore, bulk RNAseq was already shown to be able to stratify patients better than histological assessment [83]. However, scRNAseq, once the analysis becomes cheaper and more abundant, could provide even more precise patient classification into treatment groups as it can identify presence/absence of specific pathological cell subsets.

We identified several limitations in the current literature. Firstly, single-cell transcriptomics is uncovering a correlative relationship between cellular states and sustained inflammation or remission. The role and contributions of each subpopulation should be investigated in greater detail to decipher causal effects. Nevertheless, single-cell profiling can provide an excellent overview of the cell distribution in tissues, describe markers instrumental for imaging, and aid in hypothesis generation. Secondly, the selection of markers used for sorting prior to single-cell RNA sequencing can affect the subsequent analysis and some cellular populations can be unknowingly disregarded. Furthermore, different markers for identification of cells, different clustering strategies and analyses make the comparison of different datasets rather challenging. Potentially, an integrated meta-analysis comparing various datasets could verify current findings. Lastly, certain murine models of inflammatory arthritis are dependent only on an innate immune system; thus, profiling of adaptive immune cells in such cases is missing.

Single-cell transcriptomics is revolutionizing the rheumatology field. We believe that the extensive amount of literature identifying single-cell heterogeneity of the synovium will lead to better spatial characterisation of the synovium and identification of pathological interactions, which can be blocked by medical interventions to the therapeutic benefit of patients.
